# α-Mangostin hydrogel film with chitosan alginate base for recurrent aphthous stomatitis (RAS) treatment: study protocol for double-blind randomized controlled trial

**DOI:** 10.3389/fphar.2024.1353503

**Published:** 2024-02-16

**Authors:** Cszahreyloren Vitamia, Ghina Nadhifah Iftinan, Irma Rahayu Latarissa, Gofarana Wilar, Arief Cahyanto, Ahmed Fouad Abdelwahab Mohammed, Ali El-Rayyes, Nasrul Wathoni

**Affiliations:** ^1^ Doctoral Study Program, Faculty of Pharmacy, Universitas Padjadjaran, Sumedang, Indonesia; ^2^ Departement of Pharmacy, Akademi Farmasi Bumi Siliwangi, Bandung, Indonesia; ^3^ Department of Pharmaceutics and Pharmaceutical Technology, Faculty of Pharmacy, Universitas Padjadjaran, Sumedang, Indonesia; ^4^ Medication Therapy Adherence Clinic (MTAC), Universitas Padjadjaran, Sumedang, Indonesia; ^5^ Department of Pharmacology and Clinical Pharmacy, Faculty of Pharmacy, Universitas Padjadjaran, Sumedang, Indonesia; ^6^ Department of Restorative Dentistry, Faculty of Dentistry, University of Malaya, Kuala Lumpur, Malaysia; ^7^ Department of Pharmaceutics, Faculty of Pharmacy, Minia University, Minia, Egypt; ^8^ Department of Chemistry, College of Science, Northern Border University, Arar, Saudi Arabia

**Keywords:** recurrent aphthous stomatitis, α-mangostin, hydrogel film, chitosan alginate, randomized controlled trial

## Abstract

**Background:** Recurrent Aphthous Stomatitis (RAS) is a common ulcerative disease of the oral mucosa which is characterized by pain, and recurrent lesions in the oral cavity. This condition is quite painful, causing difficulty in eating, speaking and swallowing. Topical medications have been used for this condition, but the obstacle in using topical medications is the difficulty of achieving drug effects due to saliva wash out. This problem can be overcome by film hydrogel formulation which can protect the ulcer and reduce the pain to some extent. α-mangostin is a xanthone isolated from the rind of the mangosteen fruit. One of the activities of α-mangostin is anti-inflammatory effects, which operate through the characteristic mechanism of inhibiting the inflammatory response. This protocol study aims to investigate the efficacy of an α-mangostin hydrogel film with a chitosan alginate base for recurrent aphthous stomatitis (RAS) in comparison with a placebo over a period of 7 days.

**Study design:** This is a two-arm, double blinding, randomized controlled trial enrolling patients with RAS. The efficacy test of α-mangostin Hydrogel Film will be tested against the placebo. Patients with RAS will be allocated randomly into the two arms and the hydrogel film will be administered for 7 days. The diameter of ulcer and visual analog scale (VAS) score will be used as the primary efficacy endpoint. The outcome measure will be compared between the two arms at the baseline, day 3, day 5, and at the end of 7 days.

**Discussion:** The purpose of this clinical research is to provide scientific evidence on the efficacy of α-mangostin hydrogel film with a chitosan alginate basis in treating recurrent aphthous stomatitis. The trial is expected to improve our capacity to scientifically confirm the anti-inflammatory effectiveness of α-mangostin compounds in a final formulation that is ready to use.

**Trial registration:** NCT06039774 (14 September 2023).

## 1 Background

Recurrent Aphthous Stomatitis (RAS) is a common ulcerative condition affecting the oral mucosa, characterized by recurrent painful lesions in the oral cavity. This condition can significantly impact an individual’s quality of life, causing difficulties in eating, speaking, and swallowing. The etiology of RAS is not fully understood, despite numerous clinical observations and research efforts. Several factors are believed to contribute to the development of RAS, including genetic predisposition, mechanical trauma, deficiencies in micronutrients and vitamin B_12_, increased oxidative stress, food allergies, microbial factors or infections, anxiety, internal disorders, hormonal imbalances, and systemic disorders that are associated with lesions clinically similar to RAS like anemias, HIV infection, and reactive arthritis (Tarakji et al., 2015; Sharma and R, 2018; Rivera, 2019).

A cross-sectional study conducted in Shanghai, China reported that among the study population, 10.8% (11,054 individuals) were found to have oral mucosal lesions, and within this group, 1.48% suffered from RAS (Feng et al., 2015). In contrast, a separate cross-sectional study involving 4,255 North Indian individuals found an overall prevalence of RAS to be 18.93% (Kaur et al., 2021). Notably, the prevalence of RAS in the general population varies between 5% and 25% in different categories (Ślebioda, Szponar and Kowalska, 2014). It is important to mention that there is no universally standardized treatment for RAS; however, the primary objectives of all treatments are to alleviate ulcer pain, promote ulcer healing, and prevent recurrence (Sánchez-Bernal et al., 2020).

Topical medications represent the primary approach to treating RAS due to their cost-effectiveness and safety. These medications come in various forms, including liquid, solid, and semisolid preparations, such as mouthwashes, aerosols, lozenges, and ointments (Sharma et al., 2022). However, a significant challenge in using topical drugs is the limited effectiveness in achieving the desired therapeutic effects. This limitation is primarily attributed to the phenomenon of “salivary washout,” which leads to the removal and flushing of the drug from the mucosal surface. To address this issue, numerous studies have focused on enhancing the duration of drug contact with the oral mucosa. The advancement of drug delivery systems for RAS treatment is driven by three keys objectives: prolonging the residence time of the therapy at the ulcer site, delivering adequate drug concentrations to the ulcer, and alleviating pain. A promising solution entails the utilization of film formulations that act as a protective barrier for the ulcer, shielding it from external stimuli and offering relief from pain (Suharyani et al., 2021).

An oral mucoadhesive film containing chitosan was evaluated in a double-blind, randomized controlled clinical study. The findings revealed its efficacy in significantly expediting the healing process and alleviating pain in individuals afflicted with RAS. Chitosan, owing to its mucoadhesive properties, drug release characteristics, and pharmacodynamic effects, exhibits substantial potential as a drug delivery agent for the treatment of RAS (Shao and Zhou, 2020).

Herbal medicine offers an alternative approach for treating RAS by reducing pain, accelerating the healing process, and promoting overall recovery (Rezvaninejad et al., 2017). In traditional Indonesian medicine, the rind of the mangostin fruit (*Garcinia mangostana* L.) has been utilized for its therapeutic properties. α-mangostin, an xanthone compound derived from the mangosteen fruit’s rind, has been used in traditional medicine. One of its notable activities is its anti-inflammatory effect, achieved through the mechanism of inhibiting of NO, IL-1β, TNF-α, NF-κB and selective COX-2 along with blocking iNOS (Chavan and Muth, 2021). This property makes α-mangostin a promising compound for potential use in RAS management. To facilitate the delivery of α-mangostin, a combination of two mucoadhesive polymers, namely, sodium alginate and chitosan, has been employed as a drug delivery system (Mohan et al., 2018; Wathoni et al., 2019).

In a prior study, the development and assessment of α-mangostin hydrogel films with a chitosan-alginate base for *in vivo* therapy of RAS were conducted using white Wistar rats as test subjects. The results showed a remarkable 93% healing rate within 7 days (Milanda et al., 2022). As a result, further investigations will be carried out to evaluate the clinical efficacy and safety of the chitosan-alginate-based α-mangostin hydrogel film in RAS patients. This research aims to assess the suitability of the finished product for practical use in RAS treatment.

## 2 Methods

### 2.1 Study design

This is a two-arm, double-blinded, randomized controlled trial scheduled to take place at the Padjadjaran University Dental and Oral Hospital in Bandung, Indonesia. The trial aims to evaluate the efficacy and safety of α-mangostin hydrogel film in comparison to a placebo. Patients diagnosed with RAS will be randomly assigned to one of the two arms, and they will receive the mucoadhesive film for a duration of 7 days. The primary efficacy endpoints will be determined based on the measurement of ulcer diameter and the use of the Visual Analog Scale (VAS) score. These outcome measures will be assessed at various time points, including prior to the initial visit (baseline), on day 3, day 5, and at the conclusion of the 7-day treatment period based on previous trial on 2020 and 2022 (Ghorbani et al., 2020; Molania et al., 2022).

Ethics approval has been obtained from Research Ethics Committee of the Faculty of Medicine, Universitas Padjadjaran, Indonesia (approval reference number: 806/UN6.KEP/EC/2023). The trial was registered in the Clinicaltrials.gov registry (Trial number NCT06039774).

### 2.2 Study setting

The research will take place at the Padjadjaran University Dental and Oral Hospital, located in Bandung, Indonesia. Study participants will be selected from among patients diagnosed with Recurrent RAS who seek care at the oral medicine polyclinic of Padjadjaran University Dental and Oral Hospital.

### 2.3 Participants recruitment

The participants for this research project will be drawn from a pool of patients at the Padjadjaran University Dental and Oral Hospital who have been diagnosed with RAS by oral medicine specialist dentist. The participation in this study is entirely voluntary. The recruitment of patients will involve a screening process to assess their eligibility, taking into consideration the inclusion and exclusion criteria. Patient who are eligible and interested in participating in this clinical study will be provided with a detailed written informed consent and verbal explanation of the study procedures and will be randomly allocated to either the α-mangostin hydrogel film group or the placebo film group.

### 2.4 Inclusion and exclusion criteria

The efficacy test inclusion criteria include: participants aged between 18 and 59 years at the time of enrollment; patients who have been diagnosed with minor RAS from a dentist and are capable of adhering to the research protocol and providing informed consent. Eligible participants are those presenting with a minor RAS ulcer diagnosed by a dentist within 48 h of its initial appearance and who have refrained from using any medications for RAS ulcer treatment. Exclusion criteria involve individuals concurrently participating in other ongoing clinical trials.

The efficacy test exclusion criteria include: individuals with herpetiform ulcers or major aphthous ulcers; subjects with ulcers associated with systemic diseases, such as Behcet’s disease or Crohn’s disease; participants diagnosed with other severe medical conditions, including arrhythmia, uncontrolled hypertension, diabetes, hepatitis, and kidney failure; pregnant or breastfeeding women, as well as women planning to conceive during the study period. Individuals with suboptimal oral hygiene that necessitates treatment; patients with chronic illnesses requiring treatment involving antibiotics, hormones, nonsteroidal anti-inflammatory drugs, immunosuppressants, immunoenhancers, cytotoxic drugs, or cell cycle agents known to impact the oral mucosa; subjects with mental health disorders or limited cognitive capacity.

### 2.5 Sample size

Sample size was calculated with the lemeshow formula. Calculations were performed using 80% power, a 5% significance level, and 20% dropout rate. The required sample size was approximately 19 subjects for each group. We plan to enroll 24 subjects in each group to allow for a 20% withdrawal rate.

### 2.6 Randomization and blinding

A random number table will be generated using a computerized random number generator program. The resulting random allocations will be placed into opaque envelopes labeled with sequential study numbers. Two sets of these envelopes will be created: one for randomization at the site and another stored in the investigator’s office for emergency unblinding purposes. Each participant will be assigned a sequential study number, and an independent research staff member will prepare the corresponding α-mangostin hydrogel film or placebo film according to the random allocation sequence. Throughout the study, both study participants and outcome assessors will be kept unaware of the allocated intervention to maintain blinding.

### 2.7 Outcomes

Ulcer pain score, ulcer diameter and adverse event will be assessed as the outcome measurements. Ulcer pain score will be measured once a day using Visual Analog Scale (VAS) pain record individually by the participants. Ulcer diameter will be measured in the first, third, fifth, and seventh day of the study by the outcome assessors. Adverse events (AEs) will be documented once participants commence drug administration. The AE record will encompass: descriptive analysis, the timing of onset and resolution, severity, frequency, the necessity for treatment, and, if administered, the details of the treatment provided. Vital signs, including body temperature, respiratory rate, heart rate, and blood pressure, will be monitored during follow-up visits. Additionally, participants will also be queried about any discomfort symptoms they may be experiencing.

### 2.8 Intervention

**FIGURE 1:**
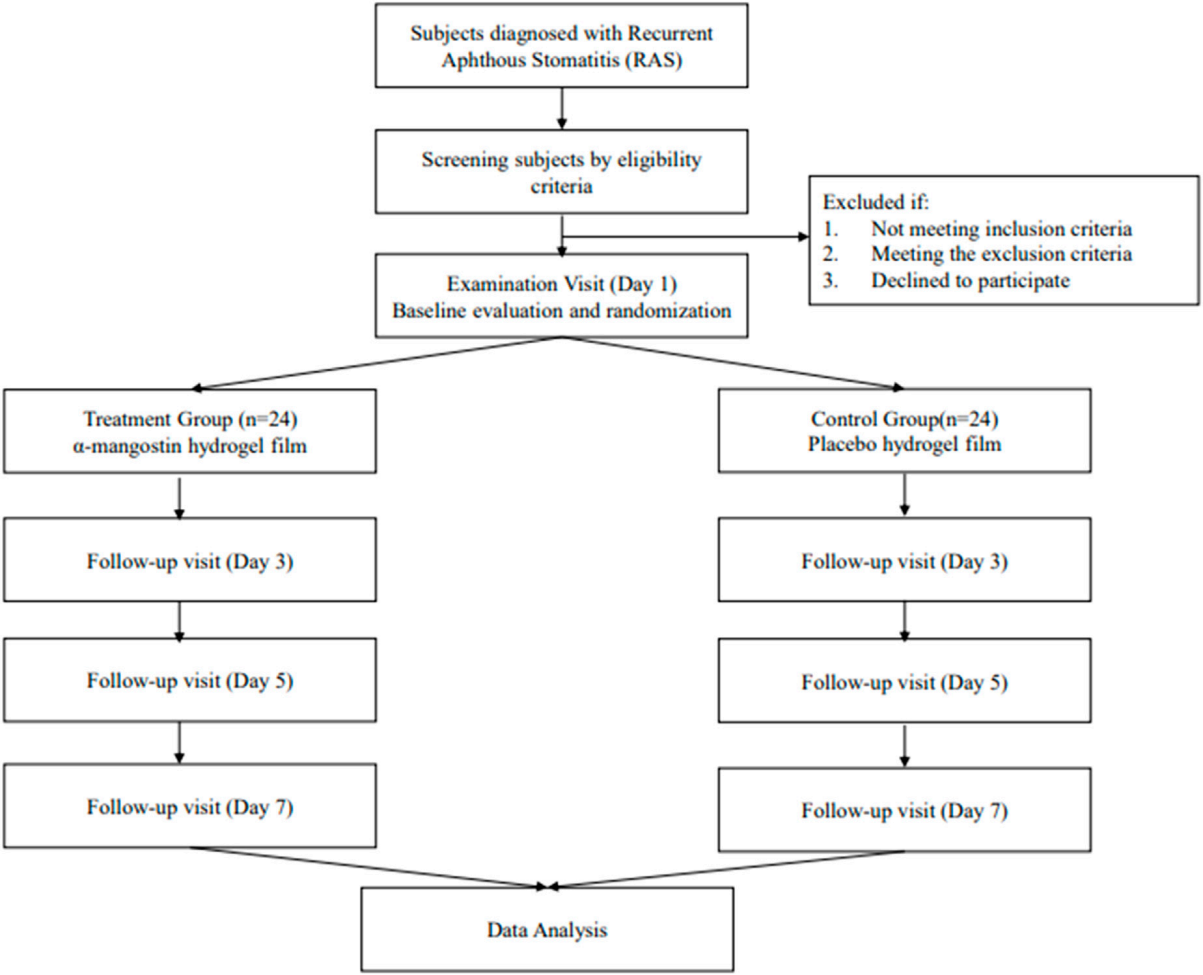
Flowchart of study protocol.

Participants will receive the α-mangostin hydrogel films with a chitosan-alginate base or a placebo hydrogel film for 7 days. Application occurs according to the following directions (Figure 1):

Patients in group 1 will receive α-mangostin hydrogel films with a chitosan-alginate base in the form of a patch. Each patch consists of 0.25 mg α-mangostin, 2% of alginate sodium, 0.2% of chitosan, 1% of glycerin, pure ethanol, and aquades. The patch is applied to the affected oral area twice daily (after breakfast and dinner) for a duration of 1 h. The patients will be advised to refrain from eating and drinking during patch application for up to 1 h post-application. In the event of a missed application of the test substance, participants are encouraged to apply the patch as soon as possible. However, if it is approaching the time for the second evening application, participant should skip the initial dose, and proceed to apply the second patch after dinner as scheduled. Participants are required to notify the researcher during evaluation and monitoring if such deviations occur. The supervision of therapy application by a pharmacist acting as the principal investigator is integral to this process.

Patients in group 2 will receive the placebo film with the same materials but without active ingredient or α-mangostin and apply with the same instructions that were provided to group 1.

The use of mouthwash, antihistamines, immunosuppressive agents, antibiotics, and analgesics is strictly prohibited during the clinical efficacy trial period. Visual analog scale records and sheets for noting side effects/adverse drug reactions are provided throughout the testing phase. Participants are instructed to record pain scores daily during the ulcerative stage and to document any side effects associated with the film usage. Ulcer size is documented on days 1 (baseline), third, fifth, and seventh. Record sheets and any remaining hydrogel film are collected at the end of the study. Participants are directed to promptly discontinue film usage if rash or oral mucositis appears during the trial and report any occurrences to the supervising physician and researcher.

### 2.9 Statistical analysis

All data will be analyzed using the Independent Samples *t*-test model in SPSS Version 26 to compare age, time of new ulcer eruption, pain scores (VAS), and ulcer size. The Chi-Square test will be employed to compare gender distribution, distribution of RAS on mucosal epithelium, history of drug allergies, and dietary habits. The significance level for statistical tests was set at α = 0.05, with a confidence level of 80% (Ghorbani et al., 2020; Shao and Zhou, 2020).

### 2.10 Data and safety monitoring

To safeguard patient privacy, all research data will be handled in line with Hospital Authority and Hospital’s policy in handling, storage, and destruction of participant’s medical record. The data will be securely stored in cabinets exclusively designated for protecting participants’ confidential information within the department. Additionally, all gathered data will be input into a computer with restricted access to investigators. The retained participant information will be stored for a period of 5 years post-study and subsequently will then undergo a secure destruction process.

## 3 Discussion

Recurrent Aphthous Stomatitis (RAS), also known as recurrent aphthous ulceration (RAU) or commonly referred to as canker sores, is a prevalent condition characterized by recurring circular ulcers that affect the oral mucosal layers. RAS entails inflammation occurring within the oral mucosal layer. This condition often arises due to hormonal changes, physical stress, trauma, alterations in psychological conditions, chemical irritations, and is even associated with allergies and genetic factors. Irritation can manifest in any part of the oral cavity, with a duration ranging from 4 to 14 days (Tarakji et al., 2015). It is recognized as one of the most painful inflammatory ulcerative conditions in the oral cavity, causing discomfort during eating, swallowing, and speaking (Moghadamnia, Motallebnejad and Khanian, 2009).

A range of medications, sourced from natural or synthetic origins, is utilized as treatments for RAS. These therapies exhibit diverse mechanisms of action, encompassing anti-inflammatory, antioxidant, immunomodulatory, analgesic, wound healing, antiulcer, antibacterial, antiviral, and antibiotic effects (Dhama et al., 2018). Alternative therapy for patients with RAS may involve the use of herbal remedies, such as α-mangostin derived from the rind of the mangosteen fruit (*Garcinia mangostana* L.). Several studies indicate that mangosteen peel extract containing α-mangostin exhibits potent antioxidant activity and the ability to promote wound healing. This suggests its potential effectiveness in formulating pharmacologically beneficial agents for wound healing (Fajeriyati, Muchtaridi and Sopyan, 2021).

A rat excision wound model was employed to investigate the effects of α-mangostin on wound healing. According to their findings, topical application of α-mangostin ointment enhanced the wound healing process. Parameters utilized in the experiment included wound closure duration and wound contraction. On the 20th day post-treatment with α-mangostin, a 90% wound healing rate was observed in the treated group. Based on their research, these results affirm that α-mangostin possesses wound healing properties (Sasikala Chinnappan et al., 2020).

Based on research, α-mangostin has gained recognition as an agent for oral cavity therapy associated with inflammatory events (Wathoni et al., 2019). In silico modeling indicates that α-mangostin exhibits the lowest binding energy with cyclooxygenase-2 (COX-2) and nuclear factor kappa-light-chain-enhancer of activated B (NFκB) proteins. α-mangostin has been found to inhibit the production of prostaglandin-E2 (PGE2), nitric oxide, and the expression of inducible nitric oxide synthase (iNOS) protein. Tumor necrosis factor-α (TNF-α) and interleukin-6 (IL-6) cytokines were significantly inhibited at concentrations of 8 and 14 g/mL. At higher doses, α-mangostin inhibits NFκB translocation while suppressing COX-2 enzyme, but not COX-1. α-mangostin inhibits total leukocyte migration, particularly *in vivo* neutrophils. Levels of TNF-α and IL-1β significantly decreased in peritoneal fluid, as measured by enzyme-linked immunosorbent assay (ELISA) analysis. Overall, these findings indicate that α-mangostin acts effectively as an anti-inflammatory agent through various mechanisms involving the inhibition of inflammation (Mohan et al., 2018).

Several previous studies also showed the use of herbal remedies to treat RAS. These treatments have shown efficacy in diminishing pain, reducing the number of ulcers, minimizing ulcer size, and expediting the healing duration. Limonene in citrus essential oil demonstrates antiulcerogenic activity. This antiulcerogenic effect of limonene functions as an immunomodulatory agent for oral aphthous ulcers. The mechanism underlying these properties is linked to its ability to boost mucus secretion, heat shock protein-70, vasoactive intestinal peptide, and prostaglandin E2 (Moraes et al., 2009). Enhancing wound healing through supportive treatments is also crucial in RAS, studies conducted in *in vitro* setting have revealed that the compound 7-O-(β-D-glucopyranosyl) galactin, present in *Ageratina pichinchensis*, has the ability to stimulate the proliferation of normal human skin cells (HFS-30) that contributes to the acceleration of wound healing (Romero-Cerecero, Zamilpa and Tortoriello, 2015). Sage extract, recognized as a natural antioxidant, has been utilized in RAS treatment. The phenolic and flavonoid compounds within sage extract have the ability to boost blood oxygen levels and safeguard the body from oxidative stress and free radicals, mitigating the risk of cell damage and providing protection against various types of ulcers (Mohammed and Abdulraheem, 2021).

The primary approach to addressing RAS involves the use of topical medications, which are deemed more cost-effective, efficient, and safe. Challenges encountered in the use of topical medications include difficulties in achieving the desired therapeutic effects. This is attributed to the common obstacle of drug delivery to the oral mucosa, known as “saliva washout,” resulting in the removal and rinsing of the drug on the mucosal surface. This issue can be addressed through the formulation of mucoadhesive film preparations, which can protect ulcers, create a barrier against external stimuli, and reduce pain to a certain extent (Suharyani et al., 2021).

Hydrogels exhibit highly favorable characteristics, including maintaining a moist environment at the wound base, absorbing minimal wound exudate, and soothing the wound surface (Zhang et al., 2018). Its insoluble hydrophilic structure demonstrates exceptional potential for absorbing wound exudate and allowing oxygen diffusion to expedite the healing process (Dhama et al., 2018). Hydrogels exhibit a distinctive capability to function as a tissue prototype, aiding in the restoration of essential components within undamaged tissue at the injury site until the process regeneration and regulation of the wound area are finalized (Dimatteo, Darling and Segura, 2018). Hydrogel films represent a three-dimensional dosage form with a hydrogel base, utilizing one or a combination of polymers to absorb and deliver active substances at the therapeutic site (Yuniarsih, Muchtaridi and Wathoni, 2018; Tran and Tran, 2019).

The hydrogel film dosage form in transdermal delivery offers several advantages, as elucidated by Yang and Pierstorff (2012) (Yang and Pierstorff, 2012):

1. Extended and Targeted Active Substance Release: Hydrogel films enable prolonged and targeted release of systemic active substances, providing better control over drug administration.
2. Enhanced Bioavailability: Hydrogel films can enhance the bioavailability of active substances by protecting them from degradation and inactivation processes in the body, thereby improving treatment effectiveness.
3. Improvement in Solubility of Poorly Soluble Drugs: Hydrogel films help improve the solubility of poorly soluble drugs in solvents, facilitating better absorption and distribution in the body.
4. Higher Treatment Cost Efficiency: By increasing drug efficacy and reducing the required dosage to achieve a specific therapeutic effect, hydrogel films can enhance the cost efficiency of treatment.
5. Reduction in Administration Frequency and Risk of Dosing Errors: Hydrogel films allow for a reduction in the frequency of drug administration, which can improve patient compliance while lowering the risk of dosing errors due to more controlled formulations.

A study demonstrated that an *in vivo* investigation of α-mangostin hydrogel films with a chitosan-alginate base for RAS therapy exhibited a favorable healing response, achieving a healing rate of 93% on the seventh day (Milanda et al., 2022). These promising outcomes have motivated us to conduct a Clinical Trial of α-mangostin Hydrogel Films with a Chitosan-Alginate Base with the aim of evaluating the clinical efficacy and safety in treating patients with RAS.

## 4 Trial status

The recruitment of participants started in December 2023 and is ongoing.

## Data Availability

The original contributions presented in the study are included in the article/Supplementary Material, further inquiries can be directed to the corresponding author.
